# Supplementary material for: Hippocampal interneuronal dysfunction and hyperexcitability in a porcine model of concussion
Source: Commun Biol. 2023 Nov 9;6:1136. doi: 10.1038/s42003-023-05491-w (PMC10636018; doi:10.1038/s42003-023-05491-w)
Supplement: Supplementary file 2 — Supplementary Figure 1 [file 42003_2023_5491_MOESM2_ESM.pdf]

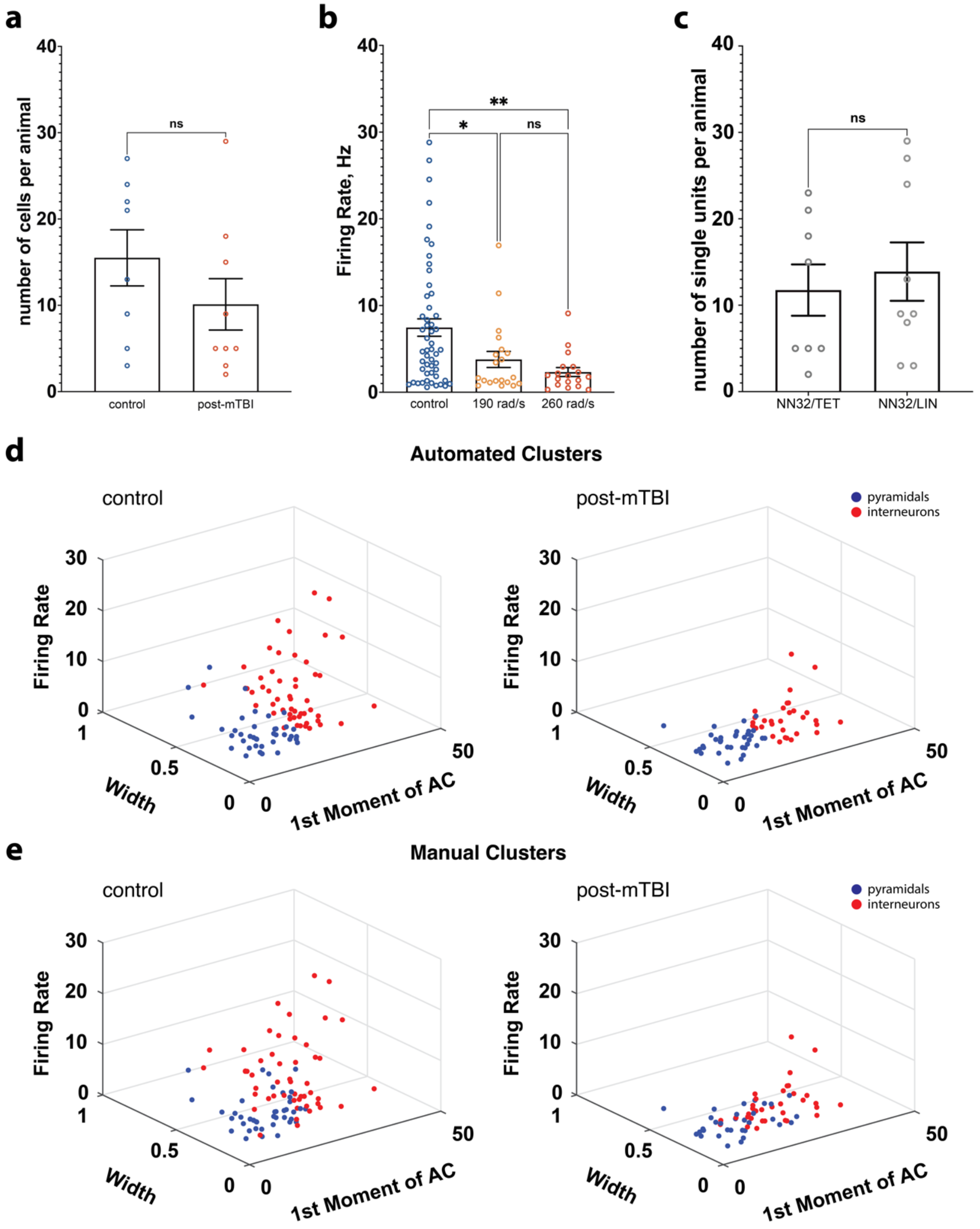

Supplementary Figure 1. Additional Single Unit Properties. a) The number of cells detected electrophysiologically were calculated in each animal and compared between the groups using unpaired t-test. There were no significant differences in the number of hippocampal cells detected in the control vs. post-mTBI group of animals (control = 16 ± 3 cells vs. post-mTBI = 10 ± 3 cells, mean ± SEM, p = 0.2397). **b)** To address the variability within the injured group due to slightly different levels of rotation (190 rad/s vs. 260 rad/s), the firing rate of CA1 interneurons was compared using ordinary one-way ANOVA analysis between the control and post-TBI groups. The firing rate decreased as a rotational velocity increased, changing significantly from 7.46 ± 1.0 Hz in control animals ($n_{animals}$ = 8, n_{cells} = 52) to 3.78 ± 0.93 Hz in animals injured at ~190 rad/sec ($n_{animals}$ = 4, n_{cells} = 20), and further to 2.33 ± 0.0.51 Hz in animals injured at ~260 rad/sec ($n_{animals}$ = 5, n_{cells} = 18, p = 0.0030). Data presented as mean ± SEM. **c)** There were no significant differences detected between a number of single units recorded with two types of electrodes (NN32/TET: $n_{animals}$ = 8, n_{cells} = 12 ± 3 cells vs. NN32/LIN: $n_{animals}$ = 8, n_{cells} = 14 ± 3 cells per animal, mean ± SEM, p = 0.6452). **d)** Automated clustering of CA1 pyramidal cells and interneurons was also performed using K-means clustering function built-in Matlab (with k = 2 clusters) and compared the results of manual clustering shown in **e).** Since an output from the manual clustering was highly similar to the automated clustering results and it had also avoided some obvious errors in subtype identification most likely due to differences between electrophysiological features of pig vs. rodent, manually identified clusters were used for the final analysis (pyramidals - blue dots, interneurons – red dots).
