# Supplementary material for: Hippocampal interneuronal dysfunction and hyperexcitability in a porcine model of concussion
Source: Commun Biol. 2023 Nov 9;6:1136. doi: 10.1038/s42003-023-05491-w (PMC10636018; doi:10.1038/s42003-023-05491-w)
Supplement: Supplementary file 3 — Reporting Summary [file 42003_2023_5491_MOESM3_ESM.pdf]

nature portfolio

Corresponding author(s): John A. Wolf, Ph.D.

Last updated by author(s): 10/06/2023

Reporting Summary

Nature Portfolio wishes to improve the reproducibility of the work that we publish. This form provides structure for consistency and transparency in reporting. For further information on Nature Portfolio policies, see our Editorial Policies and the Editorial Policy Checklist.

Statistics

For all statistical analyses, confirm that the following items are present in the figure legend, table legend, main text, or Methods section.

n/a	Confirmed	
☐	☒	The exact sample size (*n*) for each experimental group/condition, given as a discrete number and unit of measurement
☐	☒	A statement on whether measurements were taken from distinct samples or whether the same sample was measured repeatedly
☐	☒	The statistical test(s) used AND whether they are one- or two-sided<br>*Only common tests should be described solely by name; describe more complex techniques in the Methods section.*
☐	☒	A description of all covariates tested
☐	☒	A description of any assumptions or corrections, such as tests of normality and adjustment for multiple comparisons
☐	☒	A full description of the statistical parameters including central tendency (e.g. means) or other basic estimates (e.g. regression coefficient) AND variation (e.g. standard deviation) or associated estimates of uncertainty (e.g. confidence intervals)
☐	☒	For null hypothesis testing, the test statistic (e.g. *F*, *t*, *r*) with confidence intervals, effect sizes, degrees of freedom and *P* value noted<br>*Give P values as exact values whenever suitable.*
☒	☐	For Bayesian analysis, information on the choice of priors and Markov chain Monte Carlo settings
☒	☐	For hierarchical and complex designs, identification of the appropriate level for tests and full reporting of outcomes
☒	☐	Estimates of effect sizes (e.g. Cohen's *d*, Pearson's *r*), indicating how they were calculated

Our web collection on statistics for biologists contains articles on many of the points above.

Software and code

Policy information about availability of computer code

Data collection	Neurophysiological signals were amplified and acquired continuously at 32 kHz on a 64-channel Digital Lynx 4SX acquisition system with Cheetah recording and acquisition software (Neuralynx, Inc.).<br><br>A-M Systems Model 3800 stimulator with MultiStim software installed was used to generate electrical pulses, while A-M Systems Model 3820 Stimulus Isolation Unit was used to deliver the current.
Data analysis	Off-line spike detection and sorting was performed on the wideband signals using the Klusta software packages (http://klusta-team.github.io/klustakwik/), and clusters were manually refined with KlustaViewa software (https://github.com/klusta-team/klustaviewa).<br><br>Matlab version R2023a was utilized for single units' visualization and further analyses using the following freely available Matlab packages: FMAToolbox (http://fmatoolbox.sourceforge.net), Chronux (http://chronux.org) , EEGLAB (http://sccn.ucsd.edu/eeglab/index.html), and CSDplotter, version 0.1.1.<br><br>The NEURON simulation environment was utilized to model the concussion-associated electrophysiological changes observed in CA1 interneurons.<br><br>Statistical analyses were performed using Matlab version 2023a and Graphpad Prism version 10. Non-parametric Mann-Whitney test was used for analyses of neuronal properties. Two sample t-test was used for power and entrainment analyses.

For manuscripts utilizing custom algorithms or software that are central to the research but not yet described in published literature, software must be made available to editors and reviewers. We strongly encourage code deposition in a community repository (e.g. GitHub). See the Nature Portfolio guidelines for submitting code & software for further information.

Data

Policy information about availability of data

All manuscripts must include a data availability statement. This statement should provide the following information, where applicable:

- Accession codes, unique identifiers, or web links for publicly available datasets
- A description of any restrictions on data availability
- For clinical datasets or third party data, please ensure that the statement adheres to our policy

The data that support the findings of this study are available from the corresponding author upon reasonable request, but are not yet publicly available. Data are located in controlled access data storage at University of Pennsylvania.

Human research participants

Policy information about studies involving human research participants and Sex and Gender in Research.

Reporting on sex and gender	*Use the terms sex (biological attribute) and gender (shaped by social and cultural circumstances) carefully in order to avoid confusing both terms. Indicate if findings apply to only one sex or gender; describe whether sex and gender were considered in study design whether sex and/or gender was determined based on self-reporting or assigned and methods used. Provide in the source data disaggregated sex and gender data where this information has been collected, and consent has been obtained for sharing of individual-level data; provide overall numbers in this Reporting Summary. Please state if this information has not been collected. Report sex- and gender-based analyses where performed, justify reasons for lack of sex- and gender-based analysis.*
Population characteristics	*Describe the covariate-relevant population characteristics of the human research participants (e.g. age, genotypic information, past and current diagnosis and treatment categories). If you filled out the behavioural & social sciences study design questions and have nothing to add here, write "See above."*
Recruitment	*Describe how participants were recruited. Outline any potential self-selection bias or other biases that may be present and how these are likely to impact results.*
Ethics oversight	*Identify the organization(s) that approved the study protocol.*

Note that full information on the approval of the study protocol must also be provided in the manuscript.

Field-specific reporting

Please select the one below that is the best fit for your research. If you are not sure, read the appropriate sections before making your selection.

☒ Life sciences ☐ Behavioural & social sciences ☐ Ecological, evolutionary & environmental sciences

For a reference copy of the document with all sections, see nature.com/documents/nr-reporting-summary-flat.pdf

Life sciences study design

All studies must disclose on these points even when the disclosure is negative.

Sample size	Electrophysiological data were collected in 17 animals total (control = 8, post-mTBI = 9). Total number of single units detected in control (Ncells = 125) and post-mTBI (Ncells = 93) groups were used for comparison.
Data exclusions	For classification analysis of CA1 pyramidal cells vs. interneurons, the probe's anatomical location coupled with electrophysiological characteristics were utilized to exclude cells recorded at the bottom portion of the probe, as these cells could be identified as putative dentate granule cells, mossy cells, or interneurons in the dentate (control: Ncells = 14, post-mTBI: Ncells = 5). Additionally, unclassifiable cells based on their electrophysiological properties and located throughout the length of the probe were also omitted from the analysis (control: Ncells = 10, post-mTBI: Ncells = 16).
Replication	n/a
Randomization	Animals were randomly assigned to either control (n = 8) or post-mTBI (n = 9) groups.
Blinding	Most of the electrophysiological analyses were performed using automated techniques, which are blinded to the status of the animal (control vs. post-mTBI). For spike sorting analysis and neuronal classification semi-automated analyses, the independent reviewers were blinded to the status of the animals (control vs. post-mTBI).

Reporting for specific materials, systems and methods

We require information from authors about some types of materials, experimental systems and methods used in many studies. Here, indicate whether each material, system or method listed is relevant to your study. If you are not sure if a list item applies to your research, read the appropriate section before selecting a response.

Materials & experimental systems

n/a	Involved in the study
☒	☐ Antibodies
☒	☐ Eukaryotic cell lines
☒	☐ Palaeontology and archaeology
☐	☒ Animals and other organisms
☒	☐ Clinical data
☒	☐ Dual use research of concern

Methods

n/a	Involved in the study
☒	☐ ChIP-seq
☒	☐ Flow cytometry
☒	☐ MRI-based neuroimaging

Animals and other research organisms

Policy information about studies involving animals; ARRIVE guidelines recommended for reporting animal research, and Sex and Gender in Research

Laboratory animals	Adult male castrated Miniature Yucatan pigs (n = 17) were purchased from Sinclair and underwent the current studies at the age of 6 months and a mean weight of 27.7 ± 4.5 kg (mean ± std).
Wild animals	n/a
Reporting on sex	Male castrated Yucatan pigs were used in the study due to the larger brain mass at the age of the procedure, for which scaling parameters of the injury model have been established, and to remove confounds of progesterone (a neuroprotectant) and testosterone from the TBI studies. Rotational injury model necessitates the careful calibration of brain mass ratios at specific ages in order to generate injury scaling parameters that dictate specific neuropathological outcomes critical to outcome analyses described in the study. The marked differences in male versus female head / brain size in swine require complete re-characterization of the models to determine injury parameters with equivalent injury phenotype. As such, inclusion of female swine would require a considerable number of animals and effort for model re-development beyond the scope of this study.
Field-collected samples	n/a
Ethics oversight	All experiments were conducted according to the ethical guidelines set by the Institutional Animal Care and Use Committee of the University of Pennsylvania and adhered to the guidelines set forth in the NIH Public Health Service Policy on Humane Care and Use of Laboratory Animals (2015). All animals were pair housed when possible and were always in a shared room with other pigs in a research facility certified by the Association for Assessment and Accreditation of Laboratory Animal Care International (AAALAC facility).

Note that full information on the approval of the study protocol must also be provided in the manuscript.
